# The Patellostabilometer: A New Device for Quantification of Mediolateral Patella Displacement

**DOI:** 10.3390/s23031274

**Published:** 2023-01-22

**Authors:** Krzysztof Lachowski, Florian Hammermeister, Bastian Halenz, Florian Lieckefett, Thomas Götze, Robert Prill, Roland Becker

**Affiliations:** 1 Center of Orthopaedics and Traumatology, Brandenburg Medical School Theodor Fontane, University Hospital Brandenburg/Havel, 14770 Brandenburg a.d.H., Germany; 2 Technical Faculty, Brandenburg University of Applied Sciences, 14770 Brandenburg a.d.H., Germany; 3 Faculty of Health Science Brandenburg, Brandenburg Medical School Theodor Fontane, 14770 Brandenburg a.d.H., Germany

**Keywords:** patella mobility, displacement, instability, patella, patellofemoral joint, instrumented measurement, anterior knee pain, knee anatomy, measurement reliability

## Abstract

Mediolateral patella displacement is of interest for diagnostics and clinically relevant research questions. Apart from manual testing, no standardized method is currently available. Proper quantification of patella mobility is necessary to better understand pathologies at the patellofemoral joint. Patella mobility was assessed in 25 healthy individuals using a Patellostabilometer, a new prototype instrument for quantification of the mediolateral patella displacement. The participants underwent measurements of the mediolateral displacement three times using the Patellostabilometer. A maximal force of 10 N was applied for patella movement. Additionally, leg length and circumference of the knee, upper- and lower-leg were measured. Lateral patella displacement of 18.27 ± 3.76 mm (range 15.85–20.64 mm, interquartile range (IQR) of 4.79) was measured. The medial patella displacement showed 24.47 ± 6.59 mm (range 19.29–29.76 mm, IQR of 10.47). The test–retest measurement error was 2.32 ± 1.76 mm (IQR of 2.38 mm), with five outliers. There was greater test–retest variability between the measurements of the medial displacement compared to the lateral one. The test–retest variability reached 7% of the patella displacement. Other parameters provided no significant correlations. Based on the natural patellofemoral mobility, a precise and clinically relevant quantification of patella mobility is allowed.

## 1. Introduction

Both hyper- and hypomobility of the patella may become clinically symptomatic in different knee pathologies. The only method currently used as a standard and described in the literature is manual displacement testing [1]. The latest does not allow a precise estimation of mediolateral patellar mobility due to a lack of quantification of the force [2,3]. Proper quantification of patellar mobility may help to better understand pathologies at the patellofemoral joint (PFJ). There are several anatomical and histological factors which show direct impact on patella mobility, such as with the soft tissue due to the medial and lateral retinaculum, including the medial patellofemoral ligament and the iliotibial band, the bony morphology of the patellofemoral compartment, the position of the patella in regard to the trochlea groove and the quadriceps and gluteus muscle function. The patella may show hypermobility, possibly ending in patella luxation, which can occur with or without trauma. The opposite, patella hypomobility, may cause stiffness and pain [4,5]. However, it is currently difficult to define a cut off between natural and pathological patella mobility because of a lack of reference data and standardized quantification. Measurement of patellar alignment is imperative in the examination of patients with patellofemoral pain syndrome, which is reported as occurring in 25% of the population [5]. Alterations of coronal plane patellar alignment may contribute to the development of clinical conditions. Better quantification may help to identify prognostic values, which might be of therapeutic importance [6].

Several attempts have been made to define a normative range of medial and lateral patella displacement in a healthy population [3,6,7,8,9,10]. Moreover, there are some subtle forms of instability that can cause anterior knee pain or giving way phenomenon [11]. Physical examination is rather unspecific and the manual inspection alone cannot serve as an objective measurement method for assessment [1,2,3,6,10]. Nevertheless, several attempts to quantify the patellar mobility in the coronal plane were described [3,6,7,8,9,10].

For improvement of measuring the patellofemoral mobility, a more standardized method is recommended. The measurement has to be repeatable, providing a well-defined force to the patella. The force has to be well chosen, to produce movement but not to cause pain or discomfort to patients with PFJ problems. Based on these requirements, a Patellostabilometer (PSM) was developed.

The PSM was invented for quantifying the mediolateral patellar mobility in the coronal plane. Based on our clinical experience and the Kolowich’s quadrant method it was hypothesized that natural patellar mobility in both medial and lateral directions should not exceed 50% of the patella diameter in each direction [12]. The aim of our study was to evaluate the reliability of the instrumented measurement of the mediolateral displacement with the PSM in healthy patients. Such measurement should allow standardized and reproducible quantification.

## 2. Materials and Methods

### 2.1. Patellostabilometer Device

**Figure 1:**
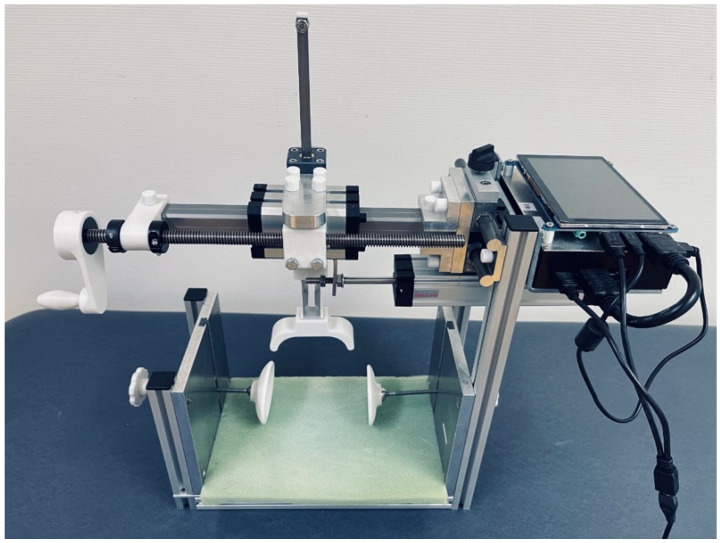
Patellostabilometer: knee pad in green, side stabilizers, grip arm and handle in white, metal crank, frame, sliders, LCD screen and the ports on the right, as described.

The PSM is a new prototype device allowing quantification of the mediolateral patella displacement (Figure 1). It is composed of a knee pad; side stabilizers, one of them removable; a grip arm on a frame; a crank with a handle and horizontal sliders. The movement of the frame on the sliders is possible in the mediolateral and craniocaudal directions. Moreover, there is an LCD screen, computer and small speaker to produce a sound signaling the amount of force, as well as ports for the power supply, video transition and USB. The latter allow the connection of a mouse, keyboard or flash disk.

There are two sensors connected to the grip arm in order to measure the amount of force applied and the displacement: a load cell and a linear potentiometer. The accuracy of the potentiometer is 0.01 mm. A load cell that emits a load-dependent signal is also installed in order to be able to measure patella mobility at a defined force. Through linearization, it is possible to convert all measurement signals into a force or mass. These parameters are saved digitally and displayed on the screen. Thereafter, those can be transferred to an USB-stick in csv file. 

### 2.2. Participants

Measurement of the patellofemoral mobility was performed in a test–retest design. Inclusion criteria were healthy males and females between 18 and 45 years of age. Exclusion criteria were patellofemoral symptoms, osteoarthritis, previous surgery, generalized ligament laxity, neuromuscular diseases, chronic pain, signs of inflammation, oedema or scars. All patients were invited to the laboratory and received a detailed explanation to ensure the most standardized test procedure possible. All patients gave written informed consent. The study was approved by the ethical committee of the university (E-02-20200405). The ethical approvement meets the institutional requirements for human experimentation in accordance with the Declaration of Helsinki.

Twenty-five healthy individuals (7 females and 18 males, age of 32.32 ± 9.29, BMI 25.22 ± 3.44) were included in this study.

### 2.3. Study Design

Additionally, leg length and circumference of the knee, upper- and lower-leg were measured. The participants underwent threefold measurements of the patellar mobility within an average interval of 11.68 ± 5.15 days.

The participants were invited one after another for the first measurement day in order to avoid longer waiting times. After information about the aim of the study, risks and data protection strategy all patients gave written informed consent. For allocating each measurement to the participant, pseudonyms were used during test procedures.

### 2.4. Measurement

The patient’s knee was assessed in a supine position. Patella mobility and ligament function were carefully assessed prior to the inclusion of the participants.

**Figure 2:**
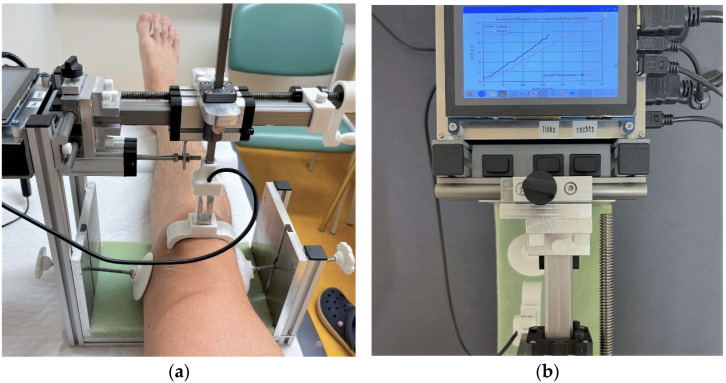
Measurement with the Patellostabilometer: (**a**) right leg correctly positioned in the device with the grip in the neutral position; (**b**) view from above at the LCD-screen, showing the acquired diagram of mediolateral mobility after a successful measurement. Red line represents the medial displacement, blue line the lateral one.

For testing, patients were positioned supine, with slight elevation of the upper trunk, and instructed not to contract their quadriceps muscles during testing. The PSM was positioned randomly, starting with the measurement either on the right or left knee first. Special caution was paid to placing the knee centrally into the device centered in reference to the mechanical axis of the lower limb and the patella. The grip arm was positioned on the patella, providing contact to the medial and lateral edge of the patella without applying any pressure towards the trochlea. Finally, the knee was stabilized due to the fixation at the medial and lateral epicondyle, preventing rotation of the leg during testing (Figure 2a). The handle for manual movement of the grip was turned clockwise to measure the lateral patella displacement and counterclockwise for the medial one.

Software for data acquisition was started and the side for measurement determined. Finally, all data were saved in an electronic file, which was downloaded later for further analysis. The sensors of the device were queried at a specific frequency; the sampling rate varied by turning the crank slowly or quickly because the output of the measured values was time-dependent. Three different acoustic signals indicated the percentages of 30%, 60% and 100% of the maximal force of 10 N. The maximal force of 10 N was chosen after pre-testing. A force–displacement graphic was shown on the display of the PSM during the testing in an online modus (Figure 2b). Raw data were collected, saved in csv format and exported via USB port to an external device. After uploading, raw data were descriptively prepared and further assessed with Microsoft Excel 365 (Microsoft Corporation, Redmond, WA, USA). For reliability, analysis IBM Statistics V.28.0.0 (IBM^®^ SPSS^®^ Statistics, Armonk, NY, USA) was used.

### 2.5. Statistics

The mean and interquartile range (IQR) are presented in mm for medial and lateral patella displacement. Mean differences and standard deviations (SD) will be presented for test–retest variability of measurement. Pearson and intraclass correlation (ICC) for a two-way mixed consistent model and Cronbachs Alpha were also analyzed [13].

## 3. Results

**Figure 3:**
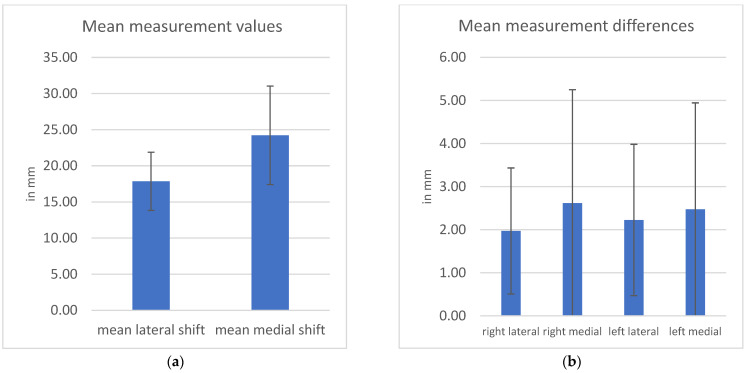
(**a**) Mean values of all measurements with standard deviation as measurement error; (**b**) mean values of differences between measurements with standard deviation as measurement error.

The lateral patella displacement was 18.27 ± 3.76 mm, with a Q1Q3 interquartile range (IQR) of (4.79) 15.85–20.64. The medial displacement was 24.47 ± 6.59 mm with an IQR of 10.47 (19.29–29.76) (Figure 3a). The test–retest measurement error was 2.32 ± 1.76 mm with an IQR of 2.38 mm, with five outliers.

The mean differences between the patellar mobility were, respectively: 1.97 ± 1.46 mm for right lateral displacement, 2.62 ± 2.63 mm for right medial displacement, 2.23 ± 1.76 mm for left lateral displacement and 2.47 ± 2.47 mm for left medial displacement (Figure 3b). The mean absolute deviation of all measurements was 1.49 ± 2.03 mm. The test–retest reliability correlation coefficients were r = 0.81, ICC(2,1) or Cronbachs Alpha = 0.89 for lateral patella displacement and r = 0.86, ICC(2,1) or Cronbachs Alpha = 0.93 for medial patella displacement.

Body height; weight; circumference of the knee, upper- and lower-leg; as well as the length proved no significant correlations (r = 0.3).

## 4. Discussion

This study showed that the PSM with the described method provides reliable measurements allowing precise quantifying of mediolateral patella displacement in healthy patients. The good and excellent non-parametric reliability coefficients for lateral and medial displacement testing support its usage for clinical examination. Therefore, our hypothesis can be accepted, because our measurement allows standardized and reproducible quantification. The variability within this group showed significant displacement differences between individuals. Further study should search for factors which may show an impact on patella mobility, such as the shape of the trochlea or patella. Data about pathologies such as patellofemoral dysplasia, MPFL injury or anterior knee problems in OA and TKA patients are also of interest. Patellofemoral mobility has been proven to have significant impact on patellofemoral pain, instability and knee stiffness and may cause functional limitations [7,14].

Medial patella displacement was larger than the lateral displacement. This observation is coherent with the literature, reporting greater medial patellar displacement due to the existence of the medial patellofemoral ligament acting as a restraint in the lateral direction. The geometry of the patellofemoral joint with a more prominent lateral trochlea is responsible for the mechanical restraint of excessive mobility. The lateral facet of the femur is often larger and extends more proximally, improving patellar stability [4,5]. The trochlea prominence is missing in patients suffering from trochlear dysplasia. The morphology of the articular facet of the patellofemoral joint is very variable, and its impact on mediolateral patella mobility needs further investigation [15].

After conducting two tests on different days, greater test–retest variability was seen between the measurements of the medial displacement compared with the lateral one. The variability of the lateral displacement was 82% only in comparison to the medial displacement. The overall divergence of patella displacement was, on average, 7%. The variability is, from the clinical point of view, considerably low [3,16]. There were five outliers, possibly caused by the intrinsic factors alternating data acquisition as described below.

Based on the natural patellofemoral mobility, a more precise quantification of patella mobility is allowed in a more standardized manner by using the PSM. Some researchers have attempted to improve the imprecise physical measurements with instrumented techniques. Reider et al. used a device mounted to the tibial tubercle to measure the mediolateral displacement during the active flexion and extension, using the weight of 9.2 kg [17]. The results of their research group indicated that during the active motion of the uninjured knee, the mean total mediolateral displacement of the patella was 22 mm, compared with 21 mm for patients with knee pain, 26 mm for patients with subluxations and 35 mm for patients with a history of patellar dislocation. However, the measurements made during the mobility of the knee are difficult to reproduce and it is doubtful if they can be used in the standardized way.

Kolowich et al. invented a quadrant method [12]. The principle of this technique was to divide the patella into equal segments in order to quantify the medial or lateral displacement as a part of the entire patellar width. The natural patella moved less than two quadrants medially or laterally when the knee was flexed to 30°, which was a different position of the knee than in the current study. However, this quadrant method corresponds to our clinical experience, in which the mediolateral patellar displacement in full extension should not exceed 50% of the patellar width. Beginning from the fully extended knee, the patella naturally translates medially, engaging the trochlear grove at 30° of flexion. There is a natural lateral patella translation up to 90° of flexion, which is accompanied by a rapidly increasing lateral patellar tilt. Another observed motion is a minimal patella rotation in the coronal plane [2,4,5,6,15,17,18]. The path that the patella follows during knee flexion is different with each individual and, according to Amis et al., this pattern does not have clinical significance [18]. The authors showed a mean difference of 6.5 mm in mediolateral patella displacement between 0° and 90° knee flexion.

In the past, stress radiographs were also used to measure the patellar displacement with medially or laterally directed forces [19]. The evaluation of the instability of the patella in the coronal plane was based on side-to-side difference. Healthy subjects showed a mean difference of less than 3.5 mm of medial and lateral displacement, while in patients after patella dislocations the lateral mean difference exceeded 10 mm. The current study of healthy subjects showed a mean difference for the lateral displacement of 2.1 mm and of 2.6 mm for the medial displacement.

In order to eliminate the constraint to patella displacement due to the prominent lateral trochlear facet, as well as the medial trochlear facet, one has to conduct the measurements in full knee extension. Whereas the patella is minimally constrained at full extension, in knee flexion the engagement of the patella in the femoral trochlea depends on the angle. The natural position of the patella may play an important role, because in full extension there is no engagement of the patella in the trochlear grove. It was reported that the patella starts to engage in the trochlear grove at about 30° flexion [17,18,19]. Thus, predominantly the medial and lateral periarticular soft tissue determines medial and lateral patella displacement, providing a physiological Caton–Deschamps index. With the measurement starting from an extended position, the medial displacement of the patella should be greater than the lateral one [19,20]. If lateral displacement exceeds the medial one, the restraints can be considered imbalanced, with a sensitivity of 91% and a specificity of 81% [20]. In contrast, in the case of patella infera, there is already a settlement in the trochlear grove and less displacement may be expected.

Apart from the bony anatomy, the mobility of the patella in the coronal plane is determined by the lateral and medial restraints, which are formed by the lateral and medial retinaculae [4,5]. There are two layers forming the lateral retinaculum: the superficial oblique retinaculum and the deep transverse ligament. The medial structures include the medial patellofemoral ligament, which originates from the adductor tubercle and inserts on the proximal 2/3 of the medial border of the patella, as well as the medial parapatellar retinaculum, which is formed from a condensation of two fascial planes on the medial aspect of the knee and which has a broad insertion on the medial patella. Another constraint is formed through the medial patellomeniscal ligament, which originates from the anterior portion of the medial meniscus and inserts onto the inferior 1/3 of the patella and the medial patellotibial ligament, which is a thickening of the anterior capsule originating on the anteromedial aspect of the tibia and inserting on the inferior aspect of the patella [5,21].

Another important aspect is the impact of the mediolateral patellar constraints on the incidence of anterior knee pain. The pathogenesis is rather multifactorial and derives from a large number of free nerve endings and fibers, particularly in the quadriceps muscles, retinacula, patellar tendon and synovium. Anterior knee pain can result from any one of these sources and clinicians typically have difficulty identifying its exact source [22,23,24]. However, patella mobility is one important factor.

There are certain factors that can influence the data acquisition. Those measurement errors can be associated with a number of intrinsic sources such as skin and fat movement, quadriceps muscle tonus or rotation of the hip. Because of this fact, there were always multiple measures taken to avoid these errors by attention to detail. The technical limitation of the PSM is the limited space between the knee pad and the grip arm, which is connected to the rigid frame. As a consequence, in cases of using the instrument on some adipose patients, patients with knee oedema or with extension deficit, those may possibly not fit into the device and will be excluded from the future studies with the PSM.

## 5. Conclusions

The force-controlled measurement of medial and lateral patella displacement using the PSM showed a high reliability. More accurate quantification of patella displacement offers new insights in the understanding of clinically relevant pathologies, which are often influenced by a combination of different passive stabilizers such as bony morphology and soft tissue properties.

## Data Availability

Data available on request due to privacy restrictions.
